# 15-Hydroxyprostaglandin dehydrogenase is upregulated by hydroxychloroquine in rheumatoid arthritis fibroblast-like synoviocytes

**DOI:** 10.3892/mmr.2015.3931

**Published:** 2015-06-15

**Authors:** HAK-JAE KIM, SORA LEE, HAW-YONG LEE, HANSOL WON, SUNG-HAE CHANG, SEONG-SU NAH

**Affiliations:** 1 Department of Clinical Pharmacology, Department of Internal Medicine, College of Medicine, Soonchunhyang University, Cheonan, Choongcheongnam-do 330-930, Republic of Korea; 2 Soonchunhyang Medical Research Institute, Department of Internal Medicine, College of Medicine, Soonchunhyang University, Cheonan, Choongcheongnam-do 330-930, Republic of Korea; 3 Division of Rheumatology, Department of Internal Medicine, College of Medicine, Soonchunhyang University, Cheonan, Choongcheongnam-do 330-930, Republic of Korea

**Keywords:** 15-hydroxyprostaglandin dehydrogenase, fibroblast-like synoviocyte, rheumatoid arthritis, hydroxychloroquine

## Abstract

15-Hydroxyprostaglandin dehydrogenase (HPGD) is the key enzyme responsible for the metabolic inactivation of prostaglandin E2 (PGE_2_) catabolism. PGE_2_ is one of the predominant catabolic factors involved in rheumatoid arthritis (RA). However, the expression and regulation of HPGD in RA fibroblast-like synoviocyte (FLS) remain to be elucidated. Disease-modifying anti-rheumatic drugs (DMARDs) are the most important anti-arthritic drugs, which reduce the effect of joint injury. The aim of the present study was to assess the expression of HPGD in RA tissues and cells, and normal synovial tissues and cells. The effect of the most popular DMARDs, hydroxychloroquine, on the expression of HPGD in RA-FLS was also investigated. Western blotting and immuno-histochemical analysis demonstrated that the expression levels of HPGD in human synovium were lower in RA synovium compared with the normal and OA synovium. In RA-FLS, the expression of HPGD was increased following treatment with several DMARDs, including sulfasalazine, methotrexate, and hydroxychloroquine. Hydroxychloroquine (10 *µ*M) treatment induced the phosphorylation of ERK, SAPK/JNK and p38. Hydroxychloroquine induced a decrease in the release of PGE_2_, which was restored by mitogen-activated protein (MAP) kinase pathway inhibitors. Hydroxychloroquine may therefore, affect the pathogenesis of RA through the MAP kinase pathway by regulating the expression of HPGD.

## Introduction

**Figure 1:**
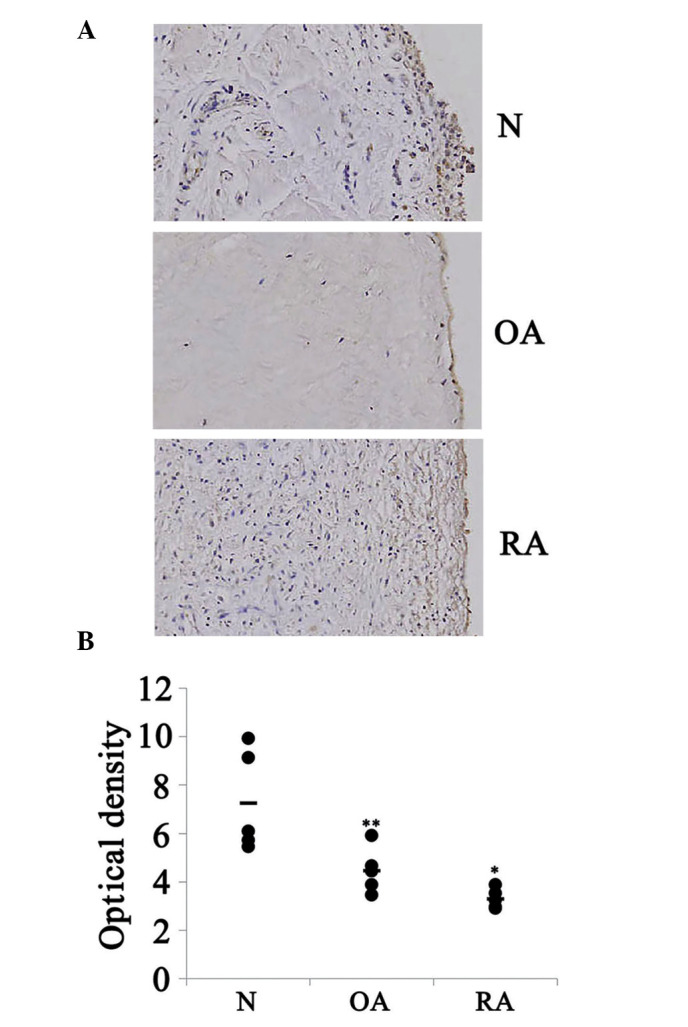
Expression of HPGD in the synovial tissue from N individuals, patients with OA and patients with RA. (A) Immunohistochemical staining revealed positive (brown) staining for HPGD in synovial tissue from N individuals, patients with OA and patients with RA (magnification, x200; n=5). (B) Quantitation of the images from using ImageJ software (^*^P<0.001, **P=0.009, compared with N). N, normal; OA, osteoarthritis; RA, rheumatoid arthritis; HPGD, 15-hydroxyprostaglandin dehydrogenase.

Rheumatoid arthritis (RA) is a chronic autoimmune disease characterized by inflammatory cell infiltration, synovial lining cell hyperplasia and hypertrophy, and a progressive destruction of cartilage and bone. Several patho-physiological mechanisms are involved in the development and progression of the disease (1). Rheumatoid joint exhibits a marked expression of synovial Prostaglandin E_2_ (PGE_2_) synthesizing enzymes, microsomal prostaglandin E2 synthase 1 (mPGES1), and cyclooxygenase (COX)1 and 2 (2). PGE_2_ is synthesized via multiple pathogenic mechanisms of RA. It acts as a mediator of pain and inflammation and promotes bone destruction (3). PGE_2_ synthesis is the result of the activities of COX and PGES. The biosynthesis and catabolism of prostaglandins is shown in Fig. 1 (4). COX1 and cytosolic PGES are constitutively expressed, whereas COX2 and mPGES1 are inducible in an inflammatory context (5). PGE_2_ is inactivated by the enzyme 15-hydroxyprostaglandindehydrogenase (HPGD) (6). HPGD is the key enzyme responsible for the biological inactivation of prostaglandins and associated eicosanoids. It has been reported that the downregulation of HPGD is associated with various types of cancer (7-10). HPGD synthesis was also found in mouse articular chondrocytes (11). However, the effect of this enzyme in the pathology of human RA remains to be elucidated.

Synthetic disease-modifying anti-rheumatic drugs (DMARDs) are a group of non-biological pharmacological agents, which can retard or inhibit the inflammatory disease process. This category comprises commonly used agents, including methotrexate (MTX), leflunomide, hydroxychloro-quine (HCQ), sulfasalazine (SSZ) and gold salts (12). HCQ is a common disease-modifying therapeutic for RA (13) and may also be used safely in long-term treatments (14). However, the effect of hydroxychloroquine on PGE_2_ metabolism and the expression of HPGD remains to be elucidated.

The present study aimed to assess the expression levels of HPGD in RA tissues and fibroblast-like synoviocytes (FLS) compared with normal tissues and to determine whether the DMARDs regulated the expression of HPGD. The signal transduction pathway of activation by one of the DMARDs in RA-FLS was determined.

## Materials and methods

### Primary cultures of human cells

Human cartilage samples were obtained from healthy individuals and patients with osteoarthritis (OA) or RA at the Soonchunhyang University Hospital (Cheonan, Korea). Primary culture was performed as previously described (15). Human articular cartilage was cut into small tissue slices and incubated and washed in medium prior to digestion with 0.1% collagenase (Invitrogen Life Technologies, Carlsbad, CA, USA) for 3 h at 37°C. Following incubation, the slices of cartilage were almost completely digested. Undigested fragments were removed by passing the solution through a nylon mesh (70 *µ*m nylon; BD Falcon, Bedford, MA, USA). The isolated cells were washed three times by centrifugation at 211 x g for 10 min and were resuspended in phosphate buffered saline (PBS; pH 7.4). Following culturing for 4 days, the cells were starved in serum-free Dulbecco's modified Eagle's medium (DMEM) with D-glucose, L-glutamine, sodium pyruvate and sodium bicarbonate, supplemented with 20% fetal bovine serum (FBS), 100 U/ml penicillin and 100 *µ*g/ml streptomycin, for 24 h at 37°C. The morphological features and the expression levels of type II collagen and aggrecan were consistent with a chondrocyte phenotype, as opposed to fibroblast-like cells. The cells were passaged upon reaching confluence by gentle trypsinization and the cells were used for experiments between passage 4 and 8.

### Immunohistochemistry

Tissue samples obtained from human synovium were fixed in 10% paraformaldehyde in PBS and were prepared using the routine method with paraffin blocks (16). To perform immunohistochemical staining for HPGD, a 9 *µ*m paraffin block was deparaffined, remoisturized and placed in 0.01 M citrate buffer solution, with subsequent heating using ultra short waves (Microwave Processor; cat. no. B35600001; Thermo Fisher Scientific, Erembodegem, Belgium) at 100°C for 20min. The tissue sections were incubated with a mixture of methanol and 0.3% H_2_O_2_ in methanol in order to remove the intrinsic peroxidase activity. The tissue sections were subsequently immunohistochemically stained using an UltraTech kit (Immunotech, Marseille, France), according to the manufacturer's instructions. The tissue sections were pretreated with 1% bovine serum albumin in PBS and were incubated with the HPGD antibody (1:100; cat. no. 160615; Cayman Chemical Co., Ann Arbor, MI, USA). Following antibody incubation, the tissue sections were incubated with biotinylate secondary antibody (1:250; cat. no. sc-2042; Santa Cruz Biotechnology, Inc., Santa Cruz, CA, USA), washed with PBS and treated with peroxidase-conjugated streptavidin. The tissue sections were then incubated with 3,3′-diaminobenzidine tetrahydro-chloride, containing 0.05% H_2_O_2_ for 3 min and counterstained with hematoxylin. Image quantification was performed with Image J software (NIH, Bethesda, MD, USA), as previously described, with minor modifications (17). Briefly, the pixel intensities in the enlarged images (magnification, x200) were calibrated by setting the display value range from 0 (black) to 255 (red). The threshold level for detection was selected by viewing histograms and adjusted to distinguish the intensity of the signal from that of the non-specific background. An identical threshold level was applied to all the images to allow valid comparison of normal, OA and RA images. The intensity of the labeling was determined using 700-pixel boxes randomly placed at different locations in the labeled area. The background intensity was determined using boxes positioned in areas of no signal.

### Immunocytochemistry

The RA-FLS were grown and subsequently incubated in a 24-well tissue culture plate (2×10^4^ cells/well) for immunofluorescence experiments. The cell culture medium was removed once the cells had grown to 80% and the cells were serum-starved for 2 h and subsequently treated with DMARDs for 24 h. The cells were washed twice with sterile PBS and were subsequently fixed with 4% paraformaldehyde in PBS for 30 min at room temperature, followed by washing twice with sterile PBS. The fixed cells were blocked in 5% horse serum for 1 h at room temperature, followed by washing twice with sterile PBS. An antibody against HPGD (1:100; Cayman Chemical, Co.) in PBS was added to the cells and incubated overnight at 4°C. The cells were washed three times with PBS and incubated with Cy3-conjugated anti-goat secondary antibody (1:500; Jackson Immuno Research Laboratories, Inc., West Grove, PA, USA) for 1 h at room temperature. The cells were mounted following washing. Fluorescence images were captured using a confocal laser scanning microscope (FV10-ASW; Olympus, Tokyo, Japan). Quantification was performed previously described, with minor modifications (17).

Briefly, the images (magnification, ×200) were analyzed using Image J software (NIH). The pixel intensity was calibrated by setting the display value ranging from 0 (black) to 255 (red). The threshold level remained constant for all the images to allow comparison of images in normal, OA and RA-FLS samples. The pixel intensity of the labeling was determined by randomly positioning boxes (300 square pixels) around the labeling at different locations on the image.

### Western blot analysis

The cells were cultured in a 10 cm culture dish to ~80% confluence (1×10^6^ cells/well) and were starved in DMEM without FBS for 24 h. The cells were subsequently incubated for 24 h in the presence of DMARDs. Wortmannin, PD98059 and SB203580 (A.G. Scientific, Inc., San Diego, CA, USA) were added to the RA-FLS 30 min prior to HCQ stimulation. Unstimulated cells were used as controls. Following stimulation with different compounds, the cells were harvested and lysed with lysis buffer, containing 1% sodium deoxycholate in 150 mM NaCl, 10 mM Hepes (pH 7.4), 0.1% sodium dodecyl sulfate (SDS), 1% Triton X-100, 5 mM ethylenediaminetetraacetic acid, 20 *µ*g/ml aprotinin, 20 *µ*g/ml leupetin and 1 mM PMSF, on ice. The lysed protein was separated on 10–20% SDS-polyacrylamide gel electrophoresis gels and transferred onto a polyvinylidene fluoride membrane. Following blocking with 5% skim milk for 1 h at room temperature, the membranes were probed with antibodies against HPGD (1:200; Cayman Chemical Co.), COX2 (1:200; Cayman Chemical Co.), COX1 (1:500; Cayman Chemical Co.), mPGES1 (1:250; Cayman Chemical Co.), phospho-ERK, p38, SAPK/JNK (1:1,000; Cell Signaling Technology, Inc., Danvers, MA, USA) and β-actin (ACTB; 1:5,000; Santa Cruz Biotechnology, Inc., Santa Cruz, CA, USA) overnight at 4°C. Following this, the membrane was washed in TBST, containing 50 mM Tris-HCl (pH 7.4), 150 mM NaCl and 0.1% Tween-20, and incubated with peroxidase-conjugated secondary antibody (1:2,000; Santa Cruz Biotechnology, Inc.) for 1 h at room temperature. The membranes were incubated with Western Bright enhanced chemilluminescence kit (Advansta, Inc., Menlo Park, CA, USA) and exposed to X-ray films. The images were captured using a ChemiDoc Imaging system (ChemiDoc™ XRS+System with Image Lab™ Software; Bio-Rad, Hercules, CA, USA). Quantitative measurements of the protein expression levels of HPGD and ACTB were performed using Image J software (NIH). The mean pixel intensities of HPGD and ACTB were measured by positioning a box around the protein band and subtracting the background intensity. The integrated density values were presented as the mean ± standard deviation between individual protein levels normalized against the integrated density value of ACTB.

### Cell viability assay

The cell viability of FLS was accessed using a 3-(4,5-dimethylthiazol-2-yl)-5-(3-carboxymethoxy phenyl)-2-(4-sulfophenyl)-2H-tetrazolium (MTS) assay kit (Promega, Madison, WI, USA), according to the manufacturer's instructions. The FLS (1×10^5^ cells/100 *µ*l/well) were grown in 96-well microtiter plates for 24 h. HCQ was added directly to the culture media and the cells were treated with various concentrations of HCQ for 24 h. The MTS cell proliferation assay reagent was added and the samples were incubated at 37°C in 5% CO_2_ for 4 h. The absorbance was measured at 490 nm using GloMax-Multi Microplate Multimode Reader (Promega), and the difference between the test and reference wavelength was calculated. The cell viability was calculated using the equation: (optical density ratio of HCQ-treated sample/non-treated sample) × 100 (%).

### Enzyme immunoassay to measure PGE_2_

The RA-FLS (1×10^5^ cells) were grown in 24-well plates and were serum starved overnight prior to stimulation with HCQ. Following washing with PBS, the cells were pretreated with HCQ at 37°C for 24 h in DMEM in an atmosphere of 5% CO_2_. The culture supernatant described above was collected at day 1. The level of PGE_2_ in the medium was determined using a PGE_2_ parameter assay kit (R&D systems, Minneapolis, MN, USA), according to the manufacturer's instructions.

### Statistical analysis

The data are presented as the mean ± standard deviation. Statistical analysis was performed using SPSS 13.0 software (SPSS Inc., Chicago, IL, USA). Groups were compared using the Student's t-test. P<0.05 was considered to indicate a statistically significant difference.

## Results

### The expression levels of HPGD in normal, OA and RA synovial tissues were analyzed by immunohistochemical staining

**Figure 2:**
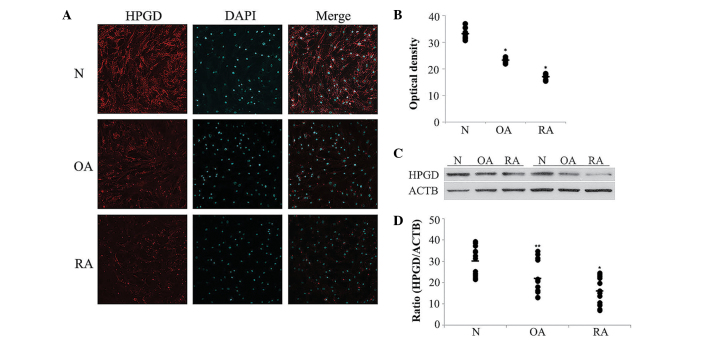
Expression of HPGD in FLS from N individuals, patients with OA and patients with RA. (A) Confocal microscopy images demonstrated immuno-histochemical staining for HPGD (red; Cy3) with DAPI, in FLS (scale bar, 50 *µ*m; n=12). (B) Quantification of the images from (A) using ImageJ software. (C) The expression of HPGD in the FLS from N individuals, patients with OA and patients with RA. ACTB was used as an internal control. (D) Graph demonstrating the ratio of HPGD to ACTB as determined by ImageJ software (^*^P<0.001, ^**^P=0.005, compared with N). N, normal; OA, osteoarthritis; RA, rheumatoid arthritis; HPGD, 15-hydroxyprostaglandin dehydrogenase; FLS, fibroblast-like synoviocytes, ACTB, β-actin.

HPGD expression in normal, OA and RA synovial tissues was analyzed by immunohistochemical staining (Fig. 1A). These images were quantified using Image J software (Fig. 1B). Immunohistochemical staining revealed that the expression levels of HPGD among the normal synovial tissue staining were marked and extensive, whereas staining in RA synovial tissue was weak and confined predominantly to the synovial membrane. In addition, inflammatory infiltrates appeared in RA synovial tissue. Normal, OA and RA-FLS were isolated from human synovial tissues. The expression levels of HPGD expression were investigated by immunofluorescence in the FLS from each synovial tissue (Fig. 2A). The immunocyto-chemical results demonstrated that the antibodies of HPGD exhibited positive reactions in normal FLS, while little fluorescence signal was observed in RA-FLS (Fig. 2B; P<0.001). The protein expression levels of HPGD in the normal, OA and RA-FLS tissues were confirmed by western blotting (Fig. 2C). Western blot analysis demonstrated that the protein expression levels of HPGD was lower in RA-FLS compared with normal and OA-FLS. An average of 1.8- and 1.3-fold less expression of HPGD was demonstrated in the RA-FLS (Fig. 2D).

**Figure 3:**
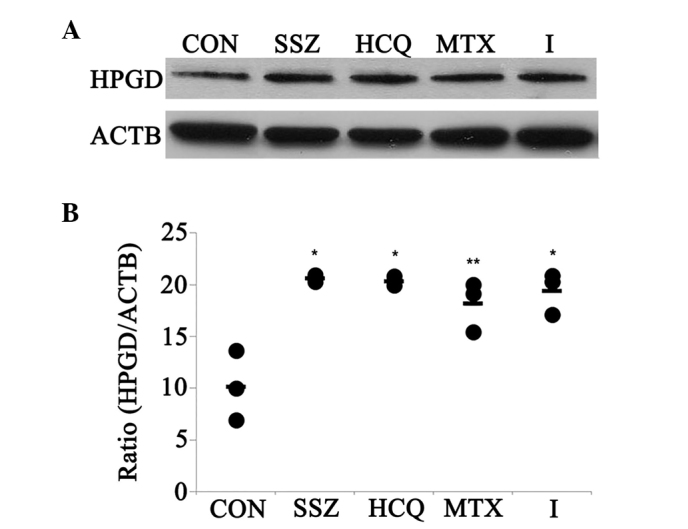
Effects of disease modifying antirheumatic drugs on the expression of HPGD in the RA-FLS. (A) The expression of HPGD in the RA-FLS was determined by western blotting (n=4). RA-FLS were serum-starved for 2 h and subsequently treated with SSZ (10 *µ*g/ml), HCQ (10 *µ*M), MTX (10 nM) or I (10 *µ*g/ml) for 24 h. (B) The graphs revealed the ratio of HPGD to ACTB, as determined by ImageJ software (^*^P≤0.001, ^**^P=0.003, compared with control). CON, control; SSZ, sulfasalazin; HCQ, hydroxychloroquine; MTX, methotrexate; I, infliximab; HPGD, 15-hydroxyprostaglandin dehydrogenase; ACTB, β-actin; RA-FLS, rheumatoid arthritis fibroblast-like synoviocytes.

Several DMARDs were assessed for their ability to induce the expression levels of HPGD in RA-FLS. The RA-FLS were cultured in a 100 mm dish up to ~80% confluence and were subsequently starved in DMEM without FBS for 2 h. The cells were incubated for 24 h in SSZ (10 *µ*g/ml), HCQ (10 *µ*M), MTX (10 nM) and infliximab (10 ug/ml). Unstimulated cells were used as controls. Western blot analysis demonstrated that all DMARDs increased the expression levels of HPGD >2-fold compared with the untreated control cells (P<0.05; Fig. 3).

**Figure 4:**
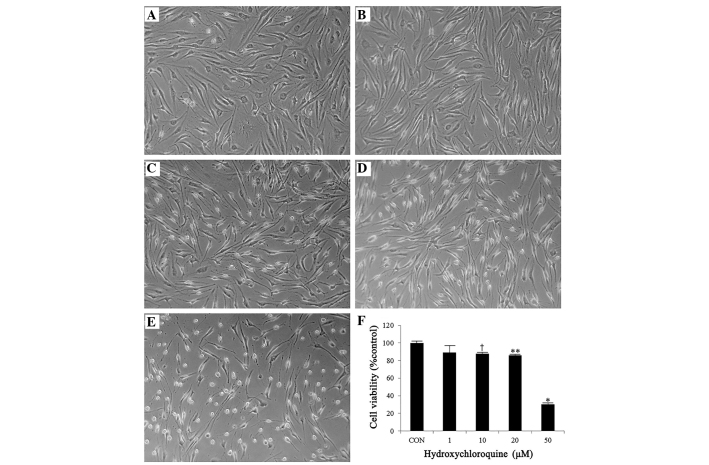
Effects of hydroxychloroquine on the viability of RA-FLS. The RA-FLS were serum-starved for 2 h and subsequently treated with different concentrations of hydroxychloroquine for 24 h. The viability of the cells was determined using an MTS assay kit. The data are shown as the mean ± standard deviation. (A) CON, (B) 1, (C) 10, (D) 20 or (E) 50 *µ*M and (F) graphs demonstrated the cell viability (38.51±2.97, ^*^P<0.001; 93.38±2.83, ^**^P=0.012; 94.78±3.31, †P=0.037, compared with the CON). CON, control; RA-FLS, rheumatoid arthritis fibroblast-like synoviocytes.

The cell viability in RA-FLS was assessed to obtain the appropriate concentration of HCQ for further experiments (Fig. 4). Cell viability was measured using an MTS assay kit, according to the manufacturer's instructions. The cells were treated with various concentrations of HCQ (1, 10, 20, and 50 *µ*M). The results demonstrated that cell viability was decreased by treatment with HCQ in a dose-dependent manner (Fig. 4A–E). Cell viability was 89.3±7.4, 87.7±1.6, 85.8±1.1 and 30.2±1.8% compared with the control at 1, 10, 20 and 50 *µ*M HCQ, respectively (Fig. 4F).

**Figure 5:**
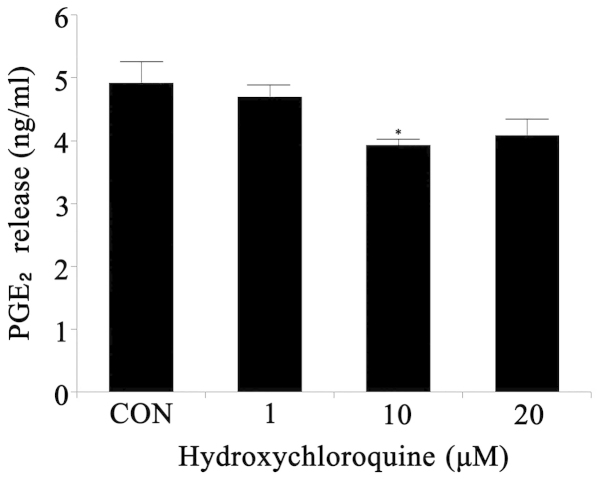
Effects of hydroxychloroquine on the release of PGE2. The RA-FLS were serum-starved for 2 h and subsequently treated with different concentrations of hydroxychloroquine for 24 h. The quantities of PGE2 and 6-keto-PGF1α were measured using a PGE2 parameter assay kit. The data are shown as the mean ± standard deviation (3.92±0.09, ^*^P=0.045, compared with the CON). PGE2, prostaglandin E2; CON, control; RA-FLS, rheumatoid arthritis fibroblast-like synoviocytes.

PGE_2_ release following HCQ stimulation in the RA-FLS was measured using an enzyme immunoassay (Fig. 5). The cells were treated with various concentrations of HCQ (1, 10, 20 and 50 *µ*M) prior to harvesting of the supernatants. The quantities of PGE_2_ and 6-keto-PGF1α, a stable metabolite of prostacyclin, were measured using a PGE_2_ parameter assay kit. PGE_2_ release was significantly decreased in the 10 *µ*M treatment group (Fig. 5).

**Figure 6:**
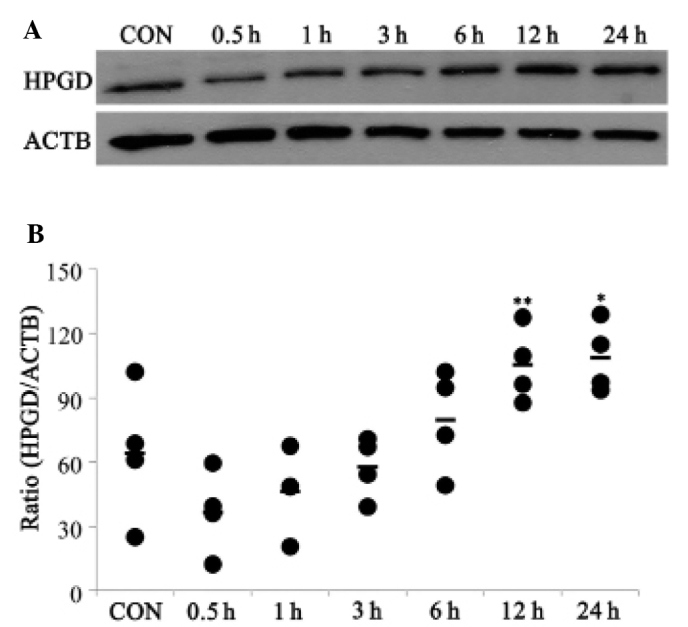
Expression of HPGD following hydroxychloroquine stimulation. The RA-FLS were serum-starved for 2 h and subsequently treated with hydroxychloroquine (10 *µ*M) for 0.5, 1, 3, 6, 12 and 24 h. (A) The expression levels of HPGD in RA-FLS was determined by immunoblotting. ACTB was used as an internal control. (B) The immunoblotting was quantified and the data are expressed as the mean ± standard deviation (111.23±16.07, ^*^P= 0.012; 106.99±16.82, ^**^P=0.019, compared with the CON; n=4). CON, control; HPGD, 15-hydroxyprostaglandin dehydrogenase; ACTB, β-actin; RA-FLS, rheumatoid arthritis fibroblast-like synoviocytes.

**Figure 7:**
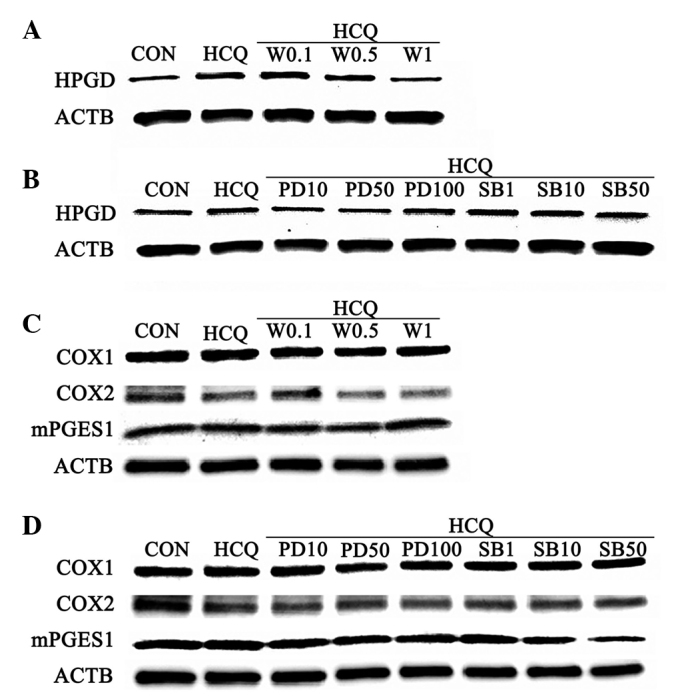
Expression levels of HPGD, COX and mPGES1 following HCQ stimulation by wortmannin, PD98059 and SB203580. The RA-FLS were serum-starved for 2 h and subsequently treated with HCQ (10 *µ*M) for 24 h. Wortmannin (0.1, 0.5 and 1 nM), PD98059 (10, 50 and 100 *µ*M) and SB203580 (1, 10 and 50 *µ*M) were added 0.5 h prior to HCQ stimulation. The expression levels of (A and B) HPGD and (C and D) COX1, COX2 and mPGES1 in RA-FLS following the different treatments. RA-FLS, rheumatoid arthritis fibroblast-like synoviocytes; CON, control; HCQ, hydroxychloroquine; W, wortmannin; PD, PD98059; SB, SB203580; HPGD, 15-hydroxyprosta-glandin dehydrogenase; ACTB, β-actin; COX, cyclooxygenase; mPGES1, microsomal prostaglandin E2 synthase 1.

**Figure 8:**
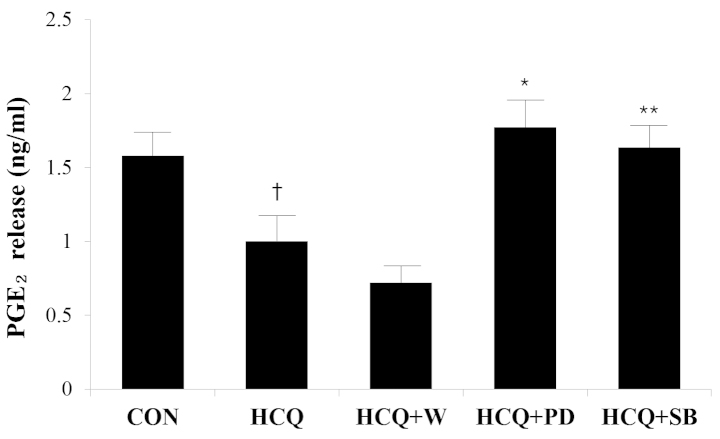
Levels of PGE2 release upon hydroxychloroquine stimulation by wortmannin, PD98059 and SB203580. The quantities of PGE2 and 6-keto-PGF1α were measured by enzyme immunoassay. Wortmannin (1 nM), PD98059 (10 *µ*M) and SB203580 (50 *µ*M) were added to the cells 0.5 h prior to HCQ (10 *µ*M) stimulation. The data are expressed as the mean ± standard deviation (1.78±0.18, ^*^P= 0.02; 1.63±0.15, ^**^P= 0.031, compared with HCQ and 1.00±0.17, ^†^P=0.050, compared with the CON). PGE2, prostaglandin E2; CON, control; HCQ, hydroxychloroquine; W, wortmannin; PD, PD98059; SB, SB203580.

**Figure 9:**
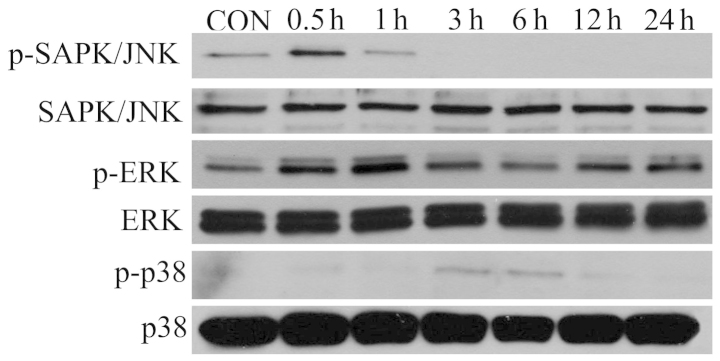
Phosphorylation of SAPK/JNK, ERK and p38 following hydroxy-chloroquine stimulation. The RA-FLS were serum-starved for 2 h and subsequently treated with hydroxychloroquine (10 *µ*M) for 0.5, 1, 3, 6, 12 and 24 h. CON, control; RA-FLS, rheumatoid arthritis fibroblast-like synovio-cytes; p, phosphorylated.

The expression levels of of HPGD following HCQ stimulation in RA-FLS were determined by western blotting. Following stimulation with HCQ (10 *µ*M), the cells were collected at different time points (0.5, 1, 3, 6, 12 and 24 h). The protein expression levels of HPGD increased in a time-dependent manner (Fig. 6). To investigate whether treatment with HCQ may affect the signal transduction pathway, the PGE_2_ metabolic enzymes were assessed by western blotting. The cells were incubated with HCQ for 24 h in the presence of several signal transduction pathway inhibitors, including Wortmannin (PI3K inhibitor), SB203580 (p38 MAP kinase inhibitor) and PD98059 (ERK inhibitor). These inhibitors were added to the RA-FLS 0.5 h prior to HCQ stimulation. It was demonstrated that the expression of HPGD was suppressed by wortmannin and PD98059 (Fig. 7A and B). However, SB203580 revealed no effect on the expression of HPGD (Fig 7B). The expression levels of COX1, COX2 and mPGES1 were assessed. The cells were incubated with HCQ for 24 h in the presence of several signal pathway inhibitors. All the inhibitors suppressed the expression levels of COX2 induced by HCQ. The expression levels of COX1 and mPGES1 were not affected by treatment with HCQ and the inhibitors (Fig. 7C and D). The level of PGE_2_ following stimulation of HCQ with inhibitors was detected by ELISA (Fig. 8). Wortmannin, PD98059 and SB203580 were added 0.5 h prior to HCQ stimulation. The decrease of HCQ-induced PGE_2_ release, was reversed by treatment with PD98059 and SB203580 (Fig. 8). Therefore, HCQ-induced PGE_2_ alterations may be associated with the MAP kinase pathway. The involvement of signal transduction pathways may be used to further assess the mechanism of stimulation of the expression of HPGD by HCQ. The expression levels of SAPK/JNK, ERK and p38 following treatment with HCQ and inhibitor-treated RA-FLS were investigated. SAPK/JNK was phosphorylated 0.5 h following HCQ stimulation (10 *µ*M) and then returned to background levels at 1 h. ERK was phosphorylated 1 h following HCQ stimulation and then returned to background levels at 3 h. p38 was phosphorylated 3 h following HCQ stimulation and the phosphorylation levels were maintained until 6 h and then returned to background levels at 12 h (Fig. 9).

## Discussion

The association between PGE_2_ metabolism and the pathology of RA is well known and the majority of previous studies have focused on PGE_2_ and the associated enzymes. However, HPGD, the enzyme responsible for the degradation of PGE_2_, has received little attention in the pathology of RA, although its expression level ultimately affects the level of PGE_2_. HPGD is the key enzyme responsible for the metabolic inactivation of PGE_2_ (18). Previous studies demonstrated a PGE_2_ mechanism involving HPGD. COX enzymes catalyze the conversion of arachidonic acid into prostaglandin H2. mPGES1 enzymes subsequently catalyze the conversion of prostaglandin H2 into PGE_2_. PGE_2_ is degraded and inactivated by the initial oxidation of their 15(S)-hydroxyl group, and this is catalyzed by HPGD (19). Previous studies have demonstrated increased quantities of COX2 and mPGES1 in OA cartilage in response to mechanical stress (5). Decreased quantities of HPGD in RA-FLS (2), eutopic endometrium (6), colonic mucosa (20) and gastric carcinoma (21) have also been reported. The expression of HPGD was absent in colonic mucosa and gastric carcinoma (20,21). Additionally, HPGD activity was absent in lung adenocarcinoma and colon cancer cells (22,23). Therefore, it is likely that the increased level of PGE_2_ in RA is a consequence of reduced catabolism and increased synthesis. Based on these previous findings, it was important to determine whether the expression levels of HPGD were changed in patients with RA. The present study demonstrated reduced expression levels of HPGD in the RA synovial tissue and FLS. The protein expression levels of HPGD were determined in RA-FLS treated with several DMARDs, to investigate whether the DMARDs affected the expression of HPGD in RA-FLS. The expression levels of HPGD were increased by treatment with DMARDs, including MTX, HCQ, SSZ and infliximab. Conventional DMARDs are generally offered as a first-line treatment for patients with RA. Biological DMARDs offer a valuable treatment alternative for patients with suboptimal response or intolerance to conventional DMARDs or when continued conventional DMARDs therapy fails (24).

HCQ, a DMARDs used in the present study, is a drug that has been used to treat autoimmune disorders, including RA and systemic lupus erythematosus (25-27). However, the specific mechanism for its pharmacological action remains to be elucidated. The present study investigated whether HCQ induced the expression of HPGD and the signal transduction pathway involved in this phenomenon. The expression levels of HPGD were increased time-dependently following treatment with HCQ (Fig. 6). Treatment with HCQ may affect the expression levels of HPGD, COX1, COX2 and mPGES1 (Fig. 7). These changes were affected by MAP-kinase inhibitors, including PD98059 and SB203580 (Fig. 7C and D). These results suggest that HCQ may affect the expression levels of HPGD via the MAP-kinase pathway in RA-FLS. In addition, these findings that PGE_2_ regulation occurs via COX2 corroborate a previous observation that HCQ negatively affected the activity of COX2 (28). Previous studies have reported that inhibitors of ERK and p38 inhibited the upregu-lation of COX2 in human follicular dendritic cells (29,30). There are increased quantities of COX2 and mPGES1 in the inflamed mucosa of inflammatory bowel disease (31,32). Otani *et al* (20) reported that the reduced expression levels of HPGD contributes to the increased levels of PGE_2_ observed in the inflamed mucosa of patients with inflammatory bowel disease. IL-4 upregulates the levels of HPGD by increasing gene transcription and decreasing protein turnover, and the upregulation can be mediated by JAK-STAT6, MAP kinases, PI3K/Akt and PKC pathways (33). The present study also demonstrated that HCQ inhibited COX2 and induced HPGD via the MAP-kinase pathway.

In conclusion, HPGD is weakly expressed in synovial tissue in conditions associated with inflammatory responses, including OA and RA. The expression levels of HPGD were lower in RA tissue compared with normal tissues. Treatment with HCQ affected the pathology of RA through the increased expression levels of HPGD and decreased levels of PGE_2_, and this may be associated with the MAP-kinase pathway. The exact role of HPGD as a potential target for the treatment of RA remains to be elucidated.
